# Hot topics in epigenetic mechanisms of aging: 2011

**DOI:** 10.1111/j.1474-9726.2012.00806.x

**Published:** 2012-04

**Authors:** María Berdasco, Manel Esteller

**Affiliations:** 1 Cancer Epigenetics and Biology Programme (PEBC), Bellvitge Biomedical Research Institute (IDIBELL)08908 L’Hospitalet de Llobregat, Barcelona, Catalonia, Spain; 2 Departament of Physiological Sciences II, School of Medicine, University of BarcelonaBarcelona, Catalonia, Spain; 3 Institució Catalana de Recerca i Estudis Avançats (ICREA)08010 Barcelona, Catalonia, Spain

**Keywords:** DNA methylation, epigenetics, histone modifications

## Abstract

Aging is a complex process that results in compromised biological functions of the organism and increased susceptibility to disease and death. Although the molecular basis of aging is currently being investigated in many experimental contexts, there is no consensus theory to fully explain the aging process. Epigenetic factors, including DNA methylation, histone modifications, and microRNA expression, may play central roles in controlling changes in gene expression and genomic instability during aging. In this Hot Topic review, we first examine the mechanisms by which these epigenetic factors contribute to aging in diverse eukaryotic species including experimental models of yeasts, worms, and mammals. In a second section, we will emphasize in the mammalian epigenetic alterations and how they may affect human longevity by altering stem cell function and/or somatic cell decline. The field of *aging epigenetics* is ripe with potential, but is still in its infancy, as new layers of complexity are emerging in the epigenetic network. As an example, we are only beginning to understand the relevance of non-coding genome to organism aging or the existence of an epigenetic memory with transgenerational inheritance. Addressing these topics will be fundamental for exploiting epigenetics phenomena as markers of aging-related diseases or as therapeutic targets.

## Introduction

Aging has been defined as a process of cellular senescence of adult tissues that results in compromised stress response, greater homeostatic imbalance, and elevated risk of disease (Rakyan *et al.*, 2010). The molecular basis of human aging is currently being investigated in many experimental contexts, including telomere shortening, DNA damage, degeneration of cell or organ structures, and changes in gene expression. Researchers have proposed that epigenetic factors, including CpG methylation, histone modifications, and non-coding RNAs (e.g., microRNAs [miRNAs]), may also be central to controlling changes in gene expression and genomic instability during aging (Mostoslavsky *et al.*, 2006; Oberdoerffer *et al.*, 2008). Although the contribution of epigenetics to several human diseases such as cancer, metabolic diseases, and neurodegenerative disorders has been proved (Berdasco & Esteller, 2010), the epigenetic variations in normal tissue owing to aging remain poorly understood. Invertebrate model organisms, such as yeast, worms, or flies, have been extensively studied in the context of longevity reporting fundamental clues about the mechanisms through epigenetic factors that contribute to aging. In contrast, the mechanisms by which epigenetics promotes aging and age-related diseases in mammals are not well defined. Several early studies investigated the epigenetic alterations of a small number of selected genes in subjects of varying age, and more recent approaches entailing similar work have employed genome-wide platforms, but there remain certain methodological limitations in selecting an optimal experimental system. In this regard, monozygotic twins are a good model for studying epigenetic changes linked to aging: they provide evidence that these changes can accumulate over time, as one individual may gradually undergo alterations that his or her twin (with identical genotype) does not (Fraga *et al.*, 2005; Schneider *et al.*, 2010). Premature aging syndromes (e.g., Hutchinson-Gilford progeria syndrome and Werner syndrome) have also been proven to be utile for studying the molecular mechanisms that contribute to mammalian aging, although we must keep in mind that these models may not fully represent the normal aging processes. Till now, several epigenetic defects have been linked to these disorders (Agrelo *et al.*, 2006; Osorio *et al.*, 2010). In sum, the field of epigenetics clearly offers great prospects for understanding of aging and of aging-related diseases such as cancer.

## Experimental models for aging studies: from yeast to humans

Epigenetic changes associated with aging could be found in a wide range of organisms going from yeast to humans. However, for most of these models, there is not a clear definition of the involvement of epigenetics and its impact on the aging processes. The only possible exception to this lack of knowledge is yeasts. For instance, it is clear that inactivation of the histone deacetylase Sir2 results in shortening of the replicative lifespan in *Sacharomyces cerevisiae,* whereas activation of Sir2 significantly extents yeast lifespan (Longo & Kennedy, 2006). Sir2 prevents aging by translocating its complex from telomeres to ribosomal DNA repeats and avoiding the formation of extrachromosomal rDNA circles (ERCs) that could result in genomic instability (Kaeberlein *et al.*, 1999). In addition, there is an increase in acetylation at H4K16 at specific subtelomeric regions associated with reduction in Sir2 expression during normal aging in yeast (Dang *et al.*, 2009). Interestingly, the levels of acetylated H4K16 in these subtelomeric regions are also controlled by the activity of Sas2, the major H4K16 acetyltransferases in yeast given rise to the hypothesis that Sas2 and Sir2 antagonistically modulate lifespan through the regulation of H4K16 acetylation (Dang *et al.*, 2009). Furthermore, a proposed role for acetylation levels at H3K56 residues has been reported (Dang *et al.*, 2009; Hachinohe *et al.*, 2011). The Sir2-associated proteins, Hst3 and Hst4, could also contribute to the genomic stabilization through their ability to directly deacetylate H3K56 and consequent heterochromatinization at ERCs (Hachinohe *et al.*, 2011).

Increased levels of Sir2 orthologs also show anti-aging effects in many other invertebrate species such as worms or flies (Tissenbaum & Guarente, 2001; Rogina & Helfand, 2004), but the connection between ERCs and aging is not clear in organisms other than yeasts. The mechanisms of lifespan extension in worms and flies seem to differ from that in yeasts. In *Caenorhabditis elegans*, increased levels of Sir2 increase lifespan in a mechanisms dependent on the insulin/IGF-1 pathway (i.e., *FOXO* transcription factors) (Tissenbaum & Guarente, 2001). The insulin/IGF pathway acts as a cascade of phosphorylation reactions that inactivates the transport of FOXO transcription factors (i.e., DAF-16) to the nucleus and the consequent inhibition of anti-aging genes such as oxidative stress or DNA damage genes. The involvement of histone methylation as a fundamental regulator of aging in worms has been also considered. The expression levels of several components of the insulin/IGF pathway in *C. elegans* have been reported to be regulated by the histone demethylase activity of UTX-1 (Jin *et al.*, 2011; Maures *et al.*, 2011). Genetic inhibition of UTX-1 increases H3K27 trimethylation of the Daf-2 gene resulting in Daf-2 downregulation but increased accumulation of Daf-16 (Jin *et al.*, 2011) and, in consequence, contributes to the activation of anti-aging genes. In addition, high levels of H3K4 trimethylation are detrimental to *C. elegans* lifespan (Greer *et al.*, 2010). Genetic defects of members of the Ash-2 complex, including the H3K4 histone methyltransferase Set2, lead to extended lifespan. In parallel, expression of the H3K4 demethylase Rbr-2 is also required for normal worm longevity, suggesting that maintenance of a H3K4 methylation level is fundamental for normal aging (Greer *et al.*, 2010). Interestingly, it is important to note that the activity of Ash-2 and Rbr-2 in worm germline seems to be fundamental for controlling aging in somatic cells (Greer *et al.*, 2010). It must be highlighted that a recent work from Brunet’s laboratory (Greer *et al.*, 2011) demonstrated a transgenerational epigenetic inheritance of longevity in worms. They found that deficiencies of the H3K4 methylation complex (including Ash-2, Wdr-5, and Set-2) in the parental generations could regulate longevity of descendants during several generations in an Rbr-2-dependent manner (Greer *et al.*, 2011). In contrast to this germline hypothesis linked to Ash-2 members, the demethylase UTX-1 regulates lifespan independently of the presence of the germline, but in a manner that depends on the insulin-FoxO signaling pathway (Maures *et al.*, 2011). More assays must be performed to investigate whether transgenerational inheritance of longevity is specific to epigenetic pathways acting during germline. In any case, these results have generated a new target for aging research: could increases of longevity (from worms to humans) be achieved by manipulation of the H3K4 methylation complex of the parents?

The study of epigenetic alterations during aging in invertebrate model organisms had produced key clues about fundamental mechanisms of mammalian aging. Although in some specific cases, we could conclude that there are conserved fundamental mechanisms of aging determination across eukaryotes, the connections are still not ascertained. Regarding sirtuins, seven mammalian Sir2 homologues (Sirt1–7) have been identified. As an example of common functions, decreased levels of the most highly related to *S. cerevisae* Sir2, Sirt1, have been observed in premature aging of mice. In contrast to yeast Sir2, which specifically deacetylates histones, Sirt1 act as a deacetylase of other proteins than histones, such as p53 acetylation (Sommer *et al.*, 2006). Like yeast Sir2, Sirt1 is able to modify chromatin and repress transcription of integrated reporter genes by means of its histone acetylase activity (Vaquero *et al.*, 2004). Similarly, disruption of Sirt6 in mice results in a degenerative phenotype with premature aging traits (including cachexia, kyphosis, and osteopenia) linked to defects in base-excision repair mechanisms (Mostoslavsky *et al.*, 2006). Recent findings suggest that the mechanism by which Sirt6 might impact on the genomic instability processes associated with aging is through its activity as an H3K9 and/or H3K56 deacetylase in telomeric regions (Michishita *et al.*, 2008; Yuan *et al.*, 2009). Furthermore, a recent genome-wide analysis of Sirt6 targets revealed that many of them are linked to aging processes through the interaction with the stress-responsive transcription factor NF-κB (Kawahara *et al.*, 2011). Other epigenetic alterations previously described in invertebrate models have been also observed in mice with premature aging phenotype. For example, Zmpste24-deficient mice (a model of Hutchinson-Gilford progeria syndrome) showed an impairment of H4K16 acetylation owing to reduced association of the histone acetyltransferase Mof to the nuclear matrix (Krishnan *et al.*, 2011). Similarly, Zmpste24-deficient mice that had been deprived of a metalloprotease involved in processing of the nuclear envelop protein lamin A and that exhibited premature aging features showed rDNA hypermethylation, which led to reduced transcription of ribosomal genes and to global hypoacetylation of histones H2B and H4 (Osorio *et al.*, 2010).

Although histone acetylation has been the main target for epigenetic studies disrupted during aging, additional studies dealing with DNA methylation and histone modifications had appeared in the last years (*see next chapters*). In this way, and similarly to worms, loss of trimethylation at H3K27 by donwregulation of the EzH2 histone methyltransferase in humans could be associated with aging-related defects, such as Hutchinson-Gilford progeria syndrome (Shumaker *et al.*, 2006). On the other hand, owing to its novelty, research on the involvement of non-coding RNA in aging deserves special attention. In the last years, several studies have been linked alterations in the expression of specific miRNAs to aging in worms (Boehm & Slack, 2005; Ibanez-Ventoso *et al.*, 2006). An overall age-related decline in miRNA expression has been described (Ibanez-Ventoso *et al.*, 2006), and increased expression of specific miRNAS (i.e., lin-4) led to extension of lifespan (Boehm & Slack, 2005). In sum, the effects of epigenetic regulation of chromatin during aging are likely to be complex, and although invertebrate and mice models had provided fundamental conclusions about basic mechanism of eukaryotic aging, the vias through they contribute overtime to somatic longevity in mammals remains poorly understood.

## Human epigenetics, aging, and stemness

A new paradigm in aging research has recently emerged: aging may also derive from a decline in the multipotent ability of adult stem cells. Adult stem cells from diverse tissues have been identified, and they contribute to tissue homeostasis by either repairing injured cells or improving tissue plasticity (Pollina & Brunet, 2011). Some tissue types have a high turnover rate, such as blood cells or gut; these present numerous adult stem cells that contribute to novel regeneration of somatic cells (e.g., erythrocyte synthesis in the blood or formation of new epithelial cells in intestinal crypts). However, in tissues with low rates of regeneration, adult stem cells may also contribute to an organism’s response to environmental factors and to tissue plasticity. Compromised adult stem cell function causes loss of tissue homeostasis and, consequently, loss of well-known cellular phenotypes in aged cells. Aging has been associated with a decreased number of stem cells, although this effect seems to depend on the tissue type and on the organism (Renault *et al.*, 2002; Nishimura *et al.*, 2005; Lugert *et al.*, 2010). Despite the altered representation of stem cells during aging, it is clear that adult stem cell function declines with age in all tissue types (Geiger & Rudolph, 2009). The dysfunction of aged stem cells may result from accumulation of irreversible modifications, including genetic alterations, mitochondrial lesions, and telomere shortening (Pollina & Brunet, 2011). Importantly, new evidence supports the notion that adult stem cell senescence and aging are also regulated by specific epigenetic modifications. In this section, we describe several relevant findings on the link between control of aged stem cells and epigenetic factors.

To asses whether the reduced differentiation capacity observed in adult stem cells during aging could derive from restriction by DNA methylation, Bocker *et al.* (2011) compared the CpG methylation profiles of hematopoietic stem cells (HPCs) acquired from umbilical cords with those of HPCs obtained from adult donors (average age: 35 years old). A bimodal pattern of differential methylation was observed: the adults exhibited hypomethylation in 350 specific CpG sites where the umbilical cord samples did not, and hypermethylation in 192 CpGs sites where the umbilical cord samples did not. Interestingly, the authors reported significant overlap between the hypomethylation pattern in these adult HPCs and that observed during myeloid differentiation (Bocker *et al.*, 2011). Furthermore, they described that age-related hypermethylation occurs in genes that are target sites of the Polycomb repressive complex 2 (PRC2), a factor that is involved in *de novo* methylation during both aging and tumorigenesis (Schlesinger *et al.*, 2007; Rakyan *et al.*, 2010). For example, the negative regulator of cytokine signaling SOCS1 involved in lymphoid differentiation became hypermethylated during aging and in multiple myeloma (Galm *et al.*, 2003), thereby revealing itself to be an important factor for pluripotency maintenance in young HPCs. A similar relationship between DNA hypermethylation and PRC2 occupancy had previously been described as a key mechanism underlying age-related methylation changes in human peripheral blood samples (Teschendorff *et al.*, 2010; Fernandez *et al.*, 2011). However, it must be noted that hypermethylation of PRC2 targets is not exclusive to stem cells; indeed, researchers have reported that in somatic cells, a subset of polycomb targets exhibit a clear trend toward hypermethylation with age, independently of disease state, sex, or cell type (upon comparison of blood, ovarian cancer, cervix and lung tissues) (Christensen *et al.*, 2009; Teschendorff *et al.*, 2010). Additionally, DNA methylation has also been implicated in replicative senescence and aging of *in vitro* experimental systems. Long-term cultured mesenchymal stem cells (long-term culture is required for large-scale *in vitro* expansion prior to therapeutic implantation) showed hypermethylation of specific CpG islands, most of which are related to homeobox genes (Bork *et al.*, 2010).

Furthermore, the role of the Polycomb group (PcG) of proteins in regulation of stem cell aging has been extensively studied. The best-known PcG regulator of adult stem cells is BMI1, a member of the Polycomb repressive complex 1 (PRC1) and a critical protein for self-renewal of blood and brain stem cells (Fasano *et al.*, 2007; Oguro *et al.*, 2010). Researchers have postulated that BMI1 controls stem cell aging throughout the regulation of important aging-related genes such as the p16^INK4a^/p19^ARF^ locus, by triggering an increase in repressive histone marks (e.g., H3K27me3) (Bracken *et al.*, 2007). Furthermore, the PcG proteins BMI1 and EzH2 are regulated by DNA methyltransferases (DNMTs) in a mechanism dependent on specific miRNA expression (So *et al.*, 2011), which adds an additional level of complexity to the epigenetic network.

Histone acetylation machinery also contributes to adult stem cell self-renewal or senescence. Loss of Sirt1 (a class III Histone deacetylase [HDAC]) enhances growth of HSCs under certain experimental conditions (Narala *et al.*, 2008). Although HDACs are known to strongly contribute to tissue homeostasis, especially in response to stress and environmental stimuli, this control remains poorly understood. For example, expression of senescence-related miRNAs, including the let-7 family, miR-23a, miR-26a, and miR-30a, could also be regulated by the activity of HDACs (Lee *et al.*, 2011). Taken together, these data clearly demonstrate that epigenetic regulatory mechanisms in adult stem cell aging overlap and cross-regulate. Nonetheless, further efforts are necessary to obtain a full overview of the connections among all the epigenetic factors and to determine whether these altered epigenetic patterns of aged stem cells cause tissue failure during organism aging.

## Epigenetic landscape of somatic differentiated cells during longevity

In addition to the fact that epigenetic alterations has been correlated with human disorders, some authors have shown that epigenetic patterns also vary in healthy cells, according to tissue type and differentiation state (Berdasco & Esteller, 2010). Thus, researchers are currently endeavoring to establish how the epigenetic landscape evolves throughout the lifetime of mammals. Some clues have already been found that support the existence of intra-individual changes in epigenetic factors during normal development and aging.

*DNA methylation*: Early evidence demonstrated that there is a global decrease in DNA methylation in different human tissues during aging (Bjornsson *et al.*, 2008). This loss was attributed to a progressive loss in DNA methylation in repetitive sequences – especially Alu elements – located throughout the genome (Bollati *et al.*, 2009). Paradoxically, and similarly to what happens in cancer, certain genes are hypermethylated. Specific age-related hypermethylation has been described at various developmentally regulated genes in various human tissues, such as *Myod1* in brain (Christensen *et al.*, 2009; Fernandez *et al.*, 2011; Hernandez *et al.*, 2011), *Pcdh10,* and *P2rx7* in intestine (Maegawa *et al.*, 2010) or *Ddah2* and *Tet2* in skin (Grönniger *et al.*, 2010). The extent of this hypermethylation is not yet known, but *de novo* methylation in skin during aging was recently found to affect < 1% of genes (Grönniger *et al.*, 2010). The aforementioned findings beg the following question: *Is there a trend to specific hypermethylation during aging?* Some authors have found significant enrichment of age-dependent CpG hypermethylation at DNA-binding factors and at transcription factors (Hernandez *et al.*, 2011), suggesting that deregulation of these so-called *master genes* could affect a broad spectrum of biological pathways and, consequently, could explain the wide phenotypic alterations of aging. However, many of the genes that were demonstrated to be hypermethylated during aging belong to the senescence and apoptosis pathways (Salminen *et al.*, 2011). Interestingly, some classic tumor-suppressor genes that are commonly hypermethylated in tumorigenesis also undergo *de novo* methylation during aging in normal tissues (Salminen *et al.*, 2011). Thus, researchers have proposed a link between hypermethylation of specific tumor-suppressor genes (e.g., LOX, p16^INK4α^, RUNX3 and TIG1) and age in non-tumorigenic gastric epithelia (So *et al.*, 2006). The three well-known, epigenetically regulated tumor-suppressor genes RARβ2, RASSF1A, and GSTP1 also become hypermethylated in premalignant prostate tissues in an age-dependent manner (Kwabi-Addo *et al.*, 2007). Likewise, the putative tumor-suppressor gene TET2 is commonly hypermethylated in myeloproliferative tumors and in aged healthy skin (Grönniger *et al.*, 2010). Whether these patterns of methylation contribute to tumorigenesis in aged tissues – namely, as markers for predisposition to acquiring genetic and/or epigenetic changes associated with tumor development – remains to be determined.

*Histone modifications:* Again, epigenetic regulation can be understood not as the consequence of a single modification (e.g., CpG methylation), but rather as the product of several epigenetic factors working in concert. Early studies showed that global levels of K20H4me3 were increased in several organs of rats older than 30 months old (Sarg *et al.*, 2002). Until this discovery was made, efforts to understand the histone modification drift during aging had developed quickly and had shifted to the area of class III HDACs (the Sirtuins). Indeed, the best example of an epigenetic change that may be linked to aging in mammals is decreased expression of sirtuin1 (SIRT1) resulting in the DNA damage-induced reorganization of chromatin (*chromatin instability*) (Sommer *et al.*, 2006; Oberdoerffer *et al.*, 2008). Most importantly, several studies on pharmacological activation of sirtuins (e.g., using resveratrol) have revealed beneficial anti-apoptotic effects (Lagouge *et al.*, 2006). The aforementioned body of evidence corroborates the awesome potential of Sirtuins as targets for anti-aging therapies.

*miRNA expression:* miRNA deregulation is an emerging and promising field in age-related epigenetics. An miRNA expression array performed in the livers of mice aged 4–33 months old showed more upregulated than downregulated miRNAs during aging (Maes *et al.*, 2008). Four miRNAs (miR-93, miR-669c, miR-214, and miR-709) were especially upregulated, and proteomic profiling of the same samples demonstrated a significant correlation between the aforementioned miRNAs and expression of the corresponding gene targets associated with mitochondrial function, oxidative stress, and proliferation (Maes *et al.*, 2008). The list of miRNAS associated with mammalian aging is rapidly increasing. Mentioning some examples: upregulation of miR-143 linked to senescence-dependent growth arrest in human fibroblasts (Bonifacio & Jarstfer, 2010), increased expression of let-7 family members in skeletal muscle aging (Drummond *et al.*, 2011), or the role of miR-27 in the aging delayed model Ames mice (Bates *et al.*, 2010). Models of premature aging, such as the *Zmpste24*-deficient mice also showed miRNA deregulation (miR-29) (Ugalde *et al.*, 2010). Interestingly, miR-29 upregulation was described also in somatic tissues from old mice during physiological aging. Increased expression is strongly associated with DNA damage and p53-pathway (Ugalde *et al.*, 2010), which would reinforce the link between aging and tumorigenesis.

## Future questions

Investigators have only just begun to explore the variation in the epigenetic landscape during aging. Numerous questions remain unanswered: *How are epigenetic changes guided to specific genes during aging? Are there specific signals that determine the exact moment of drift?* Interestingly, no significant differences in DNA methyltransferases that could explain the global decrease in DNA methylation in aged tissues have been found (Maegawa *et al.*, 2010). Moreover, the relationship between specific CpG hypermethylation in bivalent domain–enriched promoters (Rakyan *et al.*, 2010) suggests that histone modifications and chromatin remodelers may constitute a baseline for predisposing an organism’s DNA to CpG methylation. Regardless, other factors could influence DNA methylation. For example, aging-associated hypermethylation apparently depends on the CpG-island context (i.e., location, sequence type, *etc.*). Some authors have recently described a bimodal effect observed during aging in several human tissues: loci in CpG islands gained methylation with age, whereas loci outside these islands lost it (Bjornsson *et al.*, 2008; Christensen *et al.*, 2009). Additionally, the range of methylation variation depends on the gene locus: for example, imprinted genes show less extensive methylation changes during aging than do coding regions (Schneider *et al.*, 2010).

Perhaps the most important question is: *Can the epigenome be manipulated during aging?* External factors are known to contribute to epigenetic alterations during aging. For instance, several studies have shown that the DNA methylome could be directly altered by diet, xenobiotic chemicals, and exogenous stimuli (e.g., inflammation and viral/bacterial infection) (Berdasco & Esteller, 2010). The premise that nutrition alters the epigenome is especially enticing, given that it would inherently imply the possibility of reversion. Diets that are deficient in folate and methionine, which are necessary for normal biosynthesis of S-adenosylmethionine (SAM), the methyl group donor for methylcytosine, provoke DNA hypomethylation defects (Waterland *et al.*, 2006). Furthermore, the most widely studied effect of diet on aging-associated epigenetic change refers to the involvement of sirtuins in lifespan elongation mediated by caloric restriction. For example, humans who practice dietary restriction have increased levels of SIRT1, which have been strongly associated with protection against several metabolic or cardiovascular diseases. Epigenetic mechanisms are clearly linked to nutrition; furthermore, diet interventions, when applied during critical windows of development (Sandovici *et al.*, 2011), have the potential to regulate the epigenome. Thus, epigenetic players such as HDAC activators may serve as excellent targets for pharmacological treatment of human diseases.

*Can the epigenetic levels of specific genes be employed as markers of so-called bio-age?* In other words: *Is it possible to predict a person’s age based on their epigenetic status*? An exciting new application of epigenetic studies has recently been developed to answer these very questions. Bocklandt *et al.* (2011) have demonstrated that the methylation status of EDARADD, TOM1L1, and NPTX2 genes in blood samples from monozygotic twin pairs strongly correlates with their age (average accuracy: ± 5.2 years). This type of work is replete with potential applications: for example, it could ultimately benefit forensic science, and estimation of physiological age based on epigenetic markers could be employed to assess the risk that a given individual faces of acquiring certain age-related disorders.

Unfortunately, despite its promise, on the causal association between human aging and epigenetic changes remains in its infancy. Probably the greatest barriers to progress in this area are the long period of time required for assessing longevity in humans and the enormous degree of variation among individuals owing to environmental factors. To date, changes in specific epigenetic marks have been correlated with aging, but the ‘great hit’ in epigenetic control of aging will occur when we will be able to understand how epigenetic factors relate to each other. The integration of DNA modifications, histone marks, and alterations of non-coding RNAs will surely pave the way to defining reference epigenomes of aging and to identifying epigenetic alterations associated with aging and disease states.

## References

[b1] Agrelo R, Cheng WH, Setien F, Ropero S, Espada J, Fraga MF, Herranz M, Paz MF, Sanchez-Cespedes M, Artiga MJ, Guerrero D, Castells A, von Kobbe C, Bohr VA, Esteller M (2006). Epigenetic inactivation of the premature aging werner syndrome gene in human cancer. Proc. Natl Acad. Sci. USA.

[b2] Bates DJ, Li N, Liang R, Sarojini H, An J, Masternak MM, Bartke A, Wang E (2010). MicroRNA regulation in Ames dwarf mouse liver may contribute to delayed aging. Aging Cell.

[b3] Berdasco M, Esteller M (2010). Aberrant epigenetic landscape in cancer: how cellular identity goes awry. Dev. Cell.

[b4] Bjornsson HT, Sigurdsson MI, Fallin MD, Irizarry RA, Aspelund T, Cui H, Yu W, Rongione MA, Ekström TJ, Harris TB, Launer LJ, Eiriksdottir G, Leppert MF, Sapienza C, Gudnason V, Feinberg AP (2008). Intra-individual change over time in DNA methylation with familial clustering. JAMA.

[b5] Bocker MT, Hellwig I, Breiling A, Eckstein V, Ho AD, Lyko F (2011). Genome-wide promoter DNA methylation dynamics of human hematopoietic progenitor cells during differentiation and aging. Blood.

[b6] Bocklandt S, Lin W, Sehl ME, Sánchez FJ, Sinsheimer JS, Horvath S, Vilain E (2011). Epigenetic predictor of age. PLoS ONE.

[b7] Boehm M, Slack F (2005). A developmental timing microRNA and its target regulate life span in *C. elegans*. Science.

[b8] Bollati V, Schwartz J, Wright R, Litonjua A, Tarantini L, Suh H, Sparrow D, Vokonas P, Baccarelli A (2009). Decline in genomic DNA methylation through aging in a cohort of elderly subjects. Mech. Ageing Dev.

[b9] Bonifacio LN, Jarstfer MB (2010). MiRNA profile associated with replicative senescence, extended cell culture, and ectopic telomerase expression in human foreskin fibroblasts. PLoS ONE.

[b10] Bork S, Pfister S, Witt H, Horn P, Korn B, Ho AD, Wagner W (2010). DNA methylation pattern changes upon long-term culture and aging of human mesenchymal stromal cells. Aging Cell.

[b11] Bracken AP, Kleine-Kohlbrecher D, Dietrich N, Pasini D, Gargiulo G, Beekman C, Theilgaard-Mönch K, Minucci S, Porse BT, Marine JC, Hansen KH, Helin K (2007). The Polycomb group proteins bind throughout the INK4A-ARF locus and are disassociated in senescent cells. Genes Dev.

[b12] Christensen BC, Houseman EA, Marsit CJ, Zheng S, Wrensch MR, Wiemels JL, Nelson HH, Karagas MR, Padbury JF, Bueno R, Sugarbaker DJ, Yeh RF, Wiencke JK, Kelsey KT (2009). Aging and environmental exposures alter tissue-specific DNA methylation dependent upon CpG island context. PLoS Genet.

[b13] Dang W, Steffen KK, Perry R, Dorsey JA, Johnson FB, Shilatifard A, Kaeberlein M, Kennedy BK, Berger SL (2009). Histone H4 lysine 16 acetylation regulates cellular lifespan. Nature.

[b14] Drummond MJ, McCarthy JJ, Sinha M, Spratt HM, Volpi E, Esser KA, Rasmussen BB (2011). Aging and microRNA expression in human skeletal muscle: a microarray and bioinformatics analysis. Physiol. Genomics.

[b15] Fasano CA, Dimos JT, Ivanova NB, Lowry N, Lemischka IR, Temple S (2007). shRNA knockdown of BMI-1 reveals a critical role for p21-Rb pathway in NSC self-renewal during development. Cell Stem Cell.

[b16] Fernandez AF, Assenov Y, Martin-Subero JI, Balint B, Siebert R, Taniguchi H, Yamamoto H, Hidalgo M, Tan AC, Galm O, Ferrer I, Sanchez-Cespedes M, Villanueva A, Carmona J, Sanchez-Mut JV, Berdasco M, Moreno V, Capella G, Monk D, Ballestar E, Ropero S, Martinez R, Sanchez-Carbayo M, Prosper F, Agirre X, Fraga MF, Graña O, Perez-Jurado L, Mora J, Puig S, Prat J, Badimon L, Puca AA, Meltzer SJ, Lengauer T, Bridgewater J, Bock C, Esteller M (2012). A DNA methylation fingerprint of 1628 human samples. Genome Res.

[b17] Fraga MF, Ballestar E, Paz MF, Ropero S, Setien F, Ballestar ML, Heine-Suñer D, Cigudosa JC, Urioste M, Benitez J, Boix-Chornet M, Sanchez-Aguilera A, Ling C, Carlsson E, Poulsen P, Vaag A, Stephan Z, Spector TD, Wu YZ, Plass C, Esteller M (2005). Epigenetic differences arise during the lifetime of monozygotic twins. Proc. Natl Acad. Sci. USA.

[b18] Galm O, Yoshikawa H, Esteller M, Osieka R, Herman JG (2003). SOCS-1, a negative regulator of cytokine signaling, is frequently silenced by methylation in multiple myeloma. Blood.

[b19] Geiger H, Rudolph KL (2009). Aging in the lympho-hematopoietic stem cell compartment. Trends Immunol.

[b20] Greer EL, Maures TJ, Hauswirth AG, Green EM, Leeman DS, Maro GS, Han S, Banko MR, Gozani O, Brunet A (2010). Members of the H3K4 trimethylation complex regulate lifespan in a germline-dependent manner in *C. elegans*. Nature.

[b21] Greer EL, Maures TJ, Ucar D, Hauswirth AG, Mancini E, Lim JP, Bnayoun BA, Shi Y, Brunet A (2011). Transgenerational epigenetic inheritance of longevity in *Caenorhabditis elegans*. Nature.

[b22] Grönniger E, Weber B, Heil O, Peters N, Stäb F, Wenck H, Korn B, Winnefeld M, Lyko F (2010). Aging and chronic sun exposure cause distinct epigenetic changes in human skin. PLoS Genet.

[b23] Hachinohe M, Hanaoka F, Masumoto H (2011). Hst3 and Hst4 histone deacetylases regulate replicative lifespan by preventing genome instability in *Saccharomyces cerevisiae*. Genes Cells.

[b24] Hernandez DG, Nalls MA, Gibbs JR, Arepalli S, van der Brug M, Chong S, Moore M, Longo DL, Cookson MR, Traynor BJ, Singleton AB (2011). Distinct DNA methylation changes highly correlated with chronological age in the human brain. Hum. Mol. Genet.

[b25] Ibanez-Ventoso C, Yang M, Guo S, Robins H, Padgett RW, Driscoll M (2006). Modulated microRNA expression during adult lifespan in *Caenorhabditis elegans*. Aging Cell.

[b26] Jin C, Li J, Green CD, Yu X, Tang X, Han D, Xian B, Wang D, Huang X, Cao X, Yan Z, Hou L, Liu J, Shukeir N, Khaitovich P, Chen CD, Zhang H, Jenuwein T, Han JD (2011). Histone demethylase UTX-1 regulates *C. elegans* life span by targeting the insulin/IGF-1 signaling pathway. Cell Metab.

[b27] Kaeberlein M, McVey M, Guarente L (1999). The SIR2/3/4 complex and SIR2 alone promote longevity in *Saccharomyces cerevisiae* by two different mechanisms. Genes Dev.

[b28] Kawahara TL, Rapicavoli NA, Wu AR, Qu K, Quake SR, Chang HY (2011). Dynamic chromatin localization of Sirt6 shapes stress- and aging-related transcriptional networks. PLoS Genet.

[b29] Krishnan V, Chow MZ, Wang Z, Zhang L, Liu B, Liu X, Zhou Z (2011). Histone H4 lysine 16 hypoacetylation is associated with defective DNA repair and premature senescence in Zmpste24-deficient mice. Proc. Natl Acad. Sci. USA.

[b30] Kwabi-Addo B, Chung W, Shen L, Ittmann M, Wheeler T, Jelinek J, Issa JP (2007). Age-related DNA methylation changes in normal human prostate tissues. Clin. Cancer Res.

[b31] Lagouge M, Argmann C, Gerhart-Hines Z, Meziane H, Lerin C, Daussin F, Messadeq N, Milne J, Lambert P, Elliott P, Geny B, Laakso M, Puigserver P, Auwerx J (2006). Resveratrol improves mitochondrial function and protects against metabolic disease by activating SIRT1 and PGC-1alpha. Cell.

[b32] Lee S, Jung JW, Park SB, Roh K, Lee SY, Kim JH, Kang SK, Kang KS (2011). Histone deacetylase regulates high mobility group A2-targeting microRNAs in human cord blood-derived multipotent stem cell aging. Cell. Mol. Life Sci.

[b33] Longo VD, Kennedy BK (2006). Sirtuins in aging and age-related disease. Cell.

[b34] Lugert S, Basak O, Knuckles P, Haussler U, Fabel K, Götz M, Haas CA, Kempermann G, Taylor V, Giachino C (2010). Quiescent and active hippocampal neural stem cells with distinct morphologies respond selectively to physiological and pathological stimuli and aging. Cell Stem Cell.

[b35] Maegawa S, Hinkal G, Kim HS, Shen L, Zhang L, Zhang J, Zhang N, Liang S, Donehower LA, Issa JP (2010). Widespread and tissue specific age-related DNA methylation changes in mice. Genome Res.

[b36] Maes OC, An J, Sarojini H, Wang E (2008). Murine microRNAs implicated in liver functions and aging process. Mech. Ageing Dev.

[b37] Maures TJ, Greer EL, Hauswirth AG, Brunet A (2011). H3K27 demethylase UTX-1 regulates *C. elegans* lifespan in a germline-independent, insulin-dependent, manner. Aging Cell.

[b38] Michishita E, McCord RA, Berber E, Kioi M, Padilla-Nash H, Damian M, Cheung P, Kusumoto R, Kawahara TL, Barrett JC, Chang HY, Bohr VA, Ried T, Gozani O, Chua KF (2008). SIRT6 is a histone H3 lysine 9 deacetylase that modulates telomeric chromatin. Nature.

[b39] Mostoslavsky R, Chua KF, Lombard DB, Pang WW, Fischer MR, Gellon L, Liu P, Mostoslavsky G, Franco S, Murphy MM, Mills KD, Patel P, Hsu JT, Hong AL, Ford E, Cheng HL, Kennedy C, Nunez N, Bronson R, Frendewey D, Auerbach W, Valenzuela D, Karow M, Hottiger MO, Hursting S, Barrett JC, Guarente L, Mulligan R, Demple B, Yancopoulos GD, Alt FW (2006). Genomic instability and aging-like phenotype in the absence of mammalian SIRT6. Cell.

[b40] Narala SR, Allsopp RC, Wells TB, Zhang G, Prasad P, Coussens MJ, Rossi DJ, Weissman IL, Vaziri H (2008). SIRT1 acts as a nutrient-sensitive growth suppressor and its loss is associated with increased AMPK and telomerase activity. Mol. Biol. Cell.

[b41] Nishimura EK, Granter SR, Fisher DE (2005). Mechanisms of hair graying: incomplete melanocyte stem cell maintenance in the niche. Science.

[b42] Oberdoerffer P, Michan S, McVay M, Mostoslavsky R, Vann J, Park SK, Hartlerode A, Stegmuller J, Hafner A, Loerch P, Wright SM, Mills KD, Bonni A, Yankner BA, Scully R, Prolla TA, Alt FW, Sinclair DA (2008). SIRT1 redistribution on chromatin promotes genomic stability but alters gene expression during aging. Cell.

[b43] Oguro H, Yuan J, Ichikawa H, Ikawa T, Yamazaki S, Kawamoto H, Nakauchi H, Iwama A (2010). Poised lineage specification in multipotential hematopoietic stem and progenitor cells by the polycomb protein Bmi1. Cell Stem Cell.

[b44] Osorio FG, Varela I, Lara E, Puente XS, Espada J, Santoro R, Freije JM, Fraga MF, López-Otín C (2010). Nuclear envelope alterations generate an aging-like epigenetic pattern in mice deficient in Zmpste24 metalloprotease. Aging Cell.

[b45] Pollina EA, Brunet A (2011). Epigenetic regulation of aging stem cells. Oncogene.

[b46] Rakyan VK, Down TA, Maslau S, Andrew T, Yang TP, Beyan H, Whittaker P, McCann OT, Finer S, Valdes AM, Leslie RD, Deloukas P, Spector TD (2010). Human aging-associated DNA hypermethylation occurs preferentially at bivalent chromatin domains. Genome Res.

[b47] Renault V, Thornell LE, Eriksson PO, Butler-Browne G, Mouly V (2002). Regenerative potential of human skeletal muscle during aging. Aging Cell.

[b48] Rogina B, Helfand SL (2004). Sir2 mediates longevity in the fly through a pathway related to calorie restriction. Proc. Natl Acad. Sci. USA.

[b49] Salminen A, Ojala J, Kaarniranta K (2011). Apoptosis and aging: increased resistance to apoptosis enhances the aging process. Cell. Mol. Life Sci.

[b50] Sandovici I, Smith NH, Nitert MD, Ackers-Johnson M, Uribe-Lewis S, Ito Y, Jones RH, Marquez VE, Cairns W, Tadayyon M, O’Neill LP, Murrell A, Ling C, Constância M, Ozanne SE (2011). Maternal diet and aging alter the epigenetic control of a promoter-enhancer interaction at the Hnf4a gene in rat pancreatic islets. Proc. Natl Acad. Sci. USA.

[b51] Sarg B, Koutzamani E, Helliger W, Rundquist I, Lindner HH (2002). Postsynthetic trimethylation of histone H4 at lysine 20 in mammalian tissues is associated with aging. J. Biol. Chem.

[b52] Schlesinger Y, Straussman R, Keshet I, Farkash S, Hecht M, Zimmerman J, Eden E, Yakhini Z, Ben-Shushan E, Reubinoff BE, Bergman Y, Simon I, Cedar H (2007). Polycomb-mediated methylation on Lys27 of histone H3 pre-marks genes for de novo methylation in cancer. Nat. Genet.

[b53] Schneider E, Pliushch G, El Hajj N, Galetzka D, Puhl A, Schorsch M, Frauenknecht K, Riepert T, Tresch A, Müller AM, Coerdt W, Zechner U, Haaf T (2010). Spatial, temporal and interindividual epigenetic variation of functionally important DNA methylation patterns. Nucleic Acids Res.

[b54] Shumaker DK, Dechat T, Kohlmaier A, Adam SA, Bozovsky MR, Erdos MR, Eriksson M, Goldman AE, Khuon S, Collins FS, Jenuwein T, Goldman RD (2006). Mutant nuclear lamin a leads to progressive alterations of epigenetic control in premature aging. Proc. Natl Acad. Sci. USA.

[b55] So K, Tamura G, Honda T, Homma N, Waki T, Togawa N, Nishizuka S, Motoyama T (2006). Multiple tumor suppressor genes are increasingly methylated with age in non-neoplastic gastric epithelia. Cancer Sci.

[b56] So AY, Jung JW, Lee S, Kim HS, Kang KS (2011). DNA methyltransferase controls stem cell aging by regulating BMI1 and EZH2 through microRNAs. PLoS ONE.

[b57] Sommer M, Poliak N, Upadhyay S, Ratovitski E, Nelkin BD, Donehower LA, Sidransky D (2006). DeltaNp63alpha overexpression induces downregulation of Sirt1 and an accelerated aging phenotype in the mouse. Cell Cycle.

[b58] Teschendorff AE, Menon U, Gentry-Maharaj A, Ramus SJ, Weisenberger DJ, Shen H, Campan M, Noushmehr H, Bell CG, Maxwell AP, Savage DA, Mueller-Holzner E, Marth C, Kocjan G, Gayther SA, Jones A, Beck S, Wagner W, Laird PW, Jacobs IJ, Widschwendter M (2010). Age-dependent DNA methylation of genes that are suppressed in stem cells is a hallmark of cancer. Genome Res.

[b59] Tissenbaum HA, Guarente L (2001). Increased dosage of a sir-2 gene extends lifespan in *Caenorhabditis elegans*. Nature.

[b60] Ugalde AP, Ramsay AJ, de la Rosa J, Varela I, Mariño G, Cadiñanos J, Lu J, Freije JM, López-Otín C (2010). Aging and chronic DNA damage response activate a regulatory pathway involving miR-29 and p53. EMBO J.

[b61] Vaquero A, Scher M, Lee D, Erdjument-Bromage H, Tempst P, Reinberg D (2004). Human SirT1 interacts with histone H1 and promotes formation of facultative heterochromatin. Mol. Cell.

[b62] Waterland R, Lin JR, Smith CA, Jirtle RL (2006). Post-weaning diet affects genomic imprinting at the insulin-like growth factor 2 (Igf2) locus. Hum. Mol. Genet.

[b63] Yuan J, Pu M, Zhang Z, Lou Z (2009). Histone H3-K56 acetylation is important for genomic stability in mammals. Cell Cycle.

